# Architecture Optimization of a Non-Linear Autoregressive Neural Networks for Mackey-Glass Time Series Prediction Using Discrete Mycorrhiza Optimization Algorithm

**DOI:** 10.3390/mi14010149

**Published:** 2023-01-06

**Authors:** Hector Carreon-Ortiz, Fevrier Valdez, Patricia Melin, Oscar Castillo

**Affiliations:** Tijuana Institute of Technology, TecNM, Tijuana 22379, Mexico

**Keywords:** optimization, nonlinear autoregressive neural networks, Mackey–Glass

## Abstract

Recurrent Neural Networks (RNN) are basically used for applications with time series and sequential data and are currently being used in embedded devices. However, one of their drawbacks is that RNNs have a high computational cost and require the use of a significant amount of memory space. Therefore, computer equipment with a large processing capacity and memory is required. In this article, we experiment with Nonlinear Autoregressive Neural Networks (NARNN), which are a type of RNN, and we use the Discrete Mycorrhizal Optimization Algorithm (DMOA) in the optimization of the NARNN architecture. We used the Mackey-Glass chaotic time series (MG) to test the proposed approach, and very good results were obtained. In addition, some comparisons were made with other methods that used the MG and other types of Neural Networks such as Backpropagation and ANFIS, also obtaining good results. The proposed algorithm can be applied to robots, microsystems, sensors, devices, MEMS, microfluidics, piezoelectricity, motors, biosensors, 3D printing, etc.

## 1. Introduction

Optimization is not limited to applied mathematics, engineering, medicine, economics, computer science, operations research or any other science, but has become a fundamental tool in all fields, where constantly developing new algorithms and theoretical methods have allowed it to evolve in all directions, with a particular focus on artificial intelligence, such as deep learning, machine learning, computer vision, fuzzy logic systems, and quantum computing [1,2].

Optimization has grown steadily over the past 50 years. Modern society not only lives in a highly competitive environment, but is also forced to plan for growth in a sustainable manner and be concerned about resource conservation. Therefore, it is essential to optimally plan, design, operate and manage resources and assets. The first approach is to optimize each operation separately. However, the current trend is toward an integrated approach: synthesis and design, design and control, production planning, scheduling and control [3].

Theoretically, optimization has evolved to provide general solutions to linear, non-linear, unbounded and constrained network optimization problems. These optimization problems are called mathematical programming problems and are divided into two different categories: linear and nonlinear programming problems. Biologically derived genetic algorithms and simulated annealing are two equally powerful methods that have emerged in recent years. The development of computer technology has provided users with a variety of optimization codes with varying degrees of rigor and complexity. It is also possible to extend the capabilities of an existing method by integrating the features of two or more optimization methods to achieve more efficient optimization methodologies [4]; current optimization methods that can solve specific problems are still being developed, as we do not yet have a method that can solve them all, such as explained by the No Free Lunch (NFL) Algorithm [5], although the research trend is moving in that direction.

RNNs are a special class of neural network characterized by their inherent self-connectivity [6], and their variants are used in many contexts where temporal dependence of data is an important latent feature in model design [7]. The most important applications of RNNs include time series prediction [8], sequence transduction [9], language modeling [10,11,12,13], speech recognition [14], word embedding learning [15], sound modeling [16], handwriting recognition [17,18], and image generation [19]. A common variant of RNN called long short-term memory [20] is used in many of these studies.

One of the main advantages of this method with respect to others is that in general the NARNN-DMOA method is much easier to implement with better results, and with lower computation costs. Other methods use very robust Ensemble Neural Network architectures of several layers and of more than 2000 neurons and different architectures of Interval Type-2 Fuzzy Logic Systems (IT2FLSs), in addition to optimization algorithms such as PSO Genetic Algorithms [21,22,23], which implies a high computational cost.

The algorithm can be applied, as we previously mentioned, to robots, microsystems, sensors, devices, etc., in the optimization of the parameters of their models that are being experimented upon. The proposed algorithm can be used in the optimization of the architecture of a neural network or in the parameters of the membership functions of a fuzzy logic system; as we have seen in other articles [24,25,26,27], this type of experimentation with the DMOA is the subject of a future work that we plan to undertake in due course

The main contribution of this research is to use the DMOA algorithm to optimize the architecture of the NARNN neural network using the MG chaotic time series, which has not previously been done in the current literature.

The structure of this paper is as follows: (1) brief introduction of Optimization and Recurrent Neural Networks (RNNs), (2) we include a brief description of Nonlinear Autoregressive Neural Networks (NARNNN), (3) presentation of the Discrete Mycorrhiza Optimization Algorithm (DMOA) inspired by the symbiosis of plant roots and MN, (4) proposed method using the NARNN, the new DMOA algorithm and Mackey Glass chaotic time series, (5) results obtained from this research, such as statistical data, hypothesis testing and comparison of the DMOA-NARNN method with other methods, (6) in-depth discussion of the results and comparison of the error with other methods, and (7) conclusions of the obtained results.

## 2. Nonlinear Autoregressive Neural Networks

An Artificial Neural Network (ANN) is a type of neural network represented by a mathematical model inspired by the neural connections of the human brain. It is an intelligent system capable of recognizing time series patterns and nonlinear features.

Therefore, it is widely used to model nonlinear dynamic time series [28]. ANN incorporates artificial neurons to process information. It consists of single neurons connected to a network via weighted links. Each input is multiplied by a weight calculated by a mathematical function that determines the activation of the neurons. Another activation function calculates the output of the artificial neuron based on a certain threshold [29].

The output of a neuron can be written as Equation (1):

(1) $$y=f\left(b+\underset{i}{\sum }w_{i}x_{i}\right)$$

where *b* is the bias of the neuron, the bias input to the neuron algorithm is an offset value that helps the signal exceed the threshold of the activation function, *f* is the activation function, *w_i_* is the weight, *x_i_* is the input, and *y* is the output.

Several types of ANNs have been presented in the literature, including Multilayer Perceptron (MLP), in which neurons are grouped into an input layer, one or more hidden layers, and an output layer. These also include RNNs such as Layer Recurrent Networks [30], Time Delay Neural Networks (TDNN) [31], and NARNN [32]. In RNNs, the output of a dynamic system depends not only on the current inputs, but also on the history of inputs and states of the system. The NARNN is a recurrent dynamic network based on a linear autoregressive model with feedback connections, and consists of several network layers.

Humans do not start their thinking from scratch every second. As we read, we understand each word based on our understanding of the previous words. We never start thinking from scratch every time we do; our thoughts have permanence. A traditional ANN cannot do this, and it seems like a major shortcoming. For example, imagine that you want to classify what kind of event is happening at each point in a movie. It is not clear how a traditional ANN could use its reasoning about earlier events in the movie to inform later events, and RNN address this problem. They are networks with loops in them which allows information to persist.

An RNN is a type of artificial neural network that uses sequential or time series da-ta. These deep learning algorithms are commonly used for ordinal or temporal problems, such as language translation, natural language processing (NLP) [33,34], speech recognition, and image captioning [35]. They are distinguished by their "memory" because they take information from previous inputs to influence the current input and output. While traditional deep neural networks assume that inputs and outputs are independent of each other, the output of recurrent neural networks depends on previous elements within the sequence.

**Figure 1:**
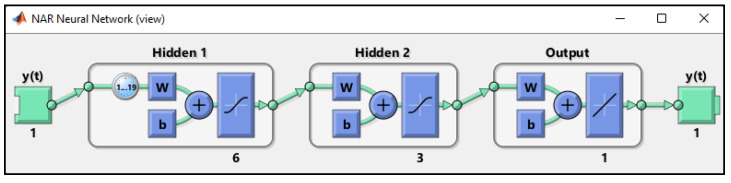
Standard NARNN schematic structure of the neural networks.

NARNNs are a type of RNN with memory and feedback capabilities. The output of each point is based on the result of the dynamic synthesis of the system before the current time. It has great advantages for modeling and simulating dynamic changes in time series [36]. Typical NARNNs mainly consist of an input layer, a hidden layer, an output layer and an input delay function, the basic structure of which is shown in Figure 1.

In Figure 1, *y(t)* is the output of the NARNN, 1..19 represents the delay order, w is the joint weight and b is the threshold of NARNNs. The model of NARNN networks can be expressed as in Equation (2), where d is the delay order and f are a nonlinear function, where the future values depend only on the previous values d of the output signal.

From the equation, it can be seen that the value of *y(t)* is determined by the values of *y(t − 1), …, y(t − d)*, which indicates that based on the continuity of data development, the model uses past values to estimate the current value [37,38].

The prediction method of the NARNN model adopts the recursive prediction method. The main purpose of this prediction method is to reproduce the predicted value one step ahead.

The future values of the time series *y(t)* are predicted only from the past values of this series. This type of prediction is called Nonlinear Autoregression (NAR) and can be written as Equation (2):

(2) $$y\left(t\right)=f\left(y\left(t-1\right),\ldots y\left(t-d\right)\right)$$

This model can be used to predict financial instruments, but it does not use additional sequences [39].

**Figure 2:**
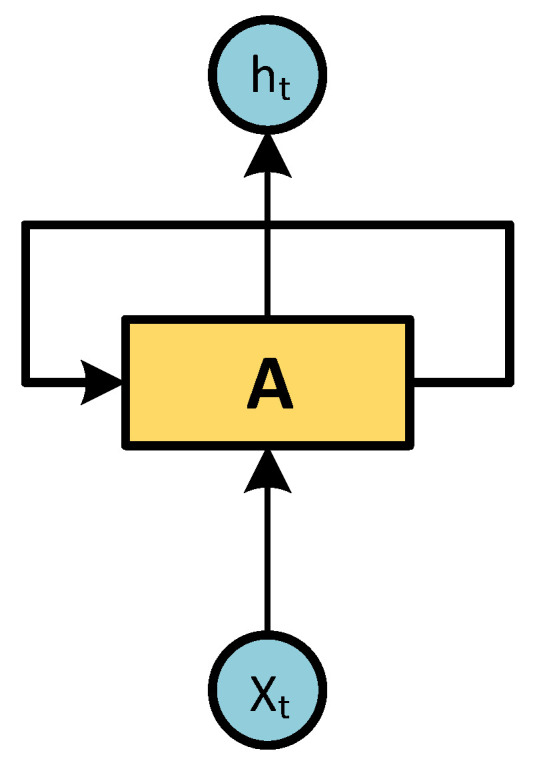
NARNN have loops.

**Figure 3:**
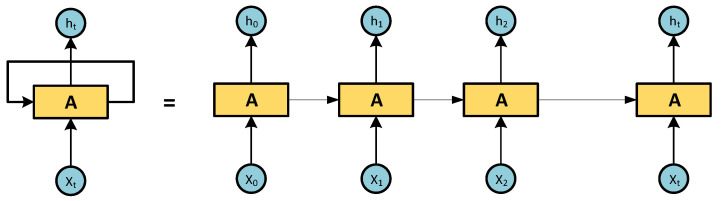
Unrolled NARNN.

Looking at Figure 2, NARNN represents the entire neural network. Figure 3 “Unrolled” represents the individual layers, or time steps, of the NARNN network. Each layer corresponds to a single piece of data [40,41].

Predicting a sequence of values in a time series is also known as multi-pass fore-casting. Closed-loop networks can perform multi-step forecasting. When external feedback is missing, closed-loop networks can still make predictions using internal feedback. In NARNN prediction, the future values of a time series are predicted only from the past values of that series.

The current literature provides a history of very extensive research on the use of NARNNs in the following areas:

- The use of NARNN in medical devices such as continuous glucose monitors and drug delivery pumps that are often combined with closed-loop systems to treat chronic diseases, for error detection and correction due to their predictive capabilities [42].
- The use of NARNNs as Chinese e-commerce sales forecasting to develop purchasing and inventory strategies for EC companies [43], to support management decisions [44], the effects of air pollution on respiratory morbidity and mortality [45], the relationship between time series in the economy [46], to model and forecast the prevalence of COVID-19 in Egypt. [47], etc.

## 3. Discrete Mycorrhiza Optimization Algorithm

Most of the world’s plant species are associated with mycorrhizal fungi in nature; this association involves the interaction of fungal hyphae on plant roots. Hyphae extend from the roots into the soil, where they absorb nutrients and transport them through the mycelium to the colonized roots [48]. Some hyphae connect host plants in what is known as a Mycorrhizal Network (MN). The MN is subway and is difficult to understand. As a result, plant and ecosystem ecologists have largely overlooked the role of MNs in plant community and ecosystem dynamics [49]. 

It is clear that most MN are present and provide nutrition to many plant species. This has important implications for plant competition for soil nutrients, seedling formation, plant succession and plant community and ecosystem dynamics [50].

Plant mycorrhizal associations have large-scale consequences throughout the eco-system [51,52]. The origins of plant-fungal symbiosis are ancient and have been proposed as a mechanism to facilitate soil colonization by plants 400 Mya [53,54]. Mycorrhizal symbiosis is a many-to-many relationship: plants tend to form symbioses with a diverse set of fungal species and, similarly, fungal species tend to be able to colonize plants of different species [55].

**Figure 4:**
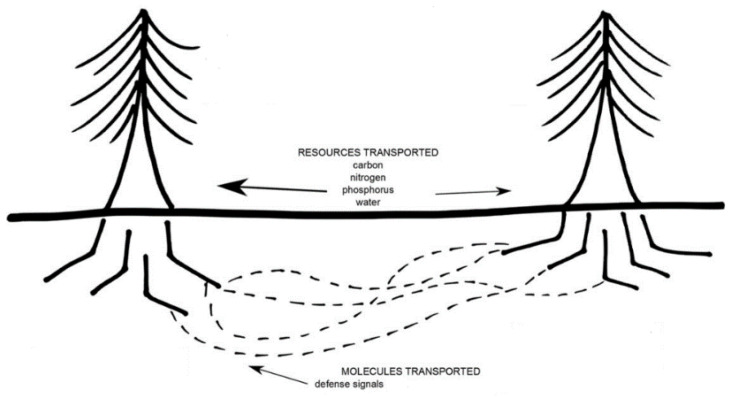
Signaling and resource exchange through MN.

**Figure 5:**
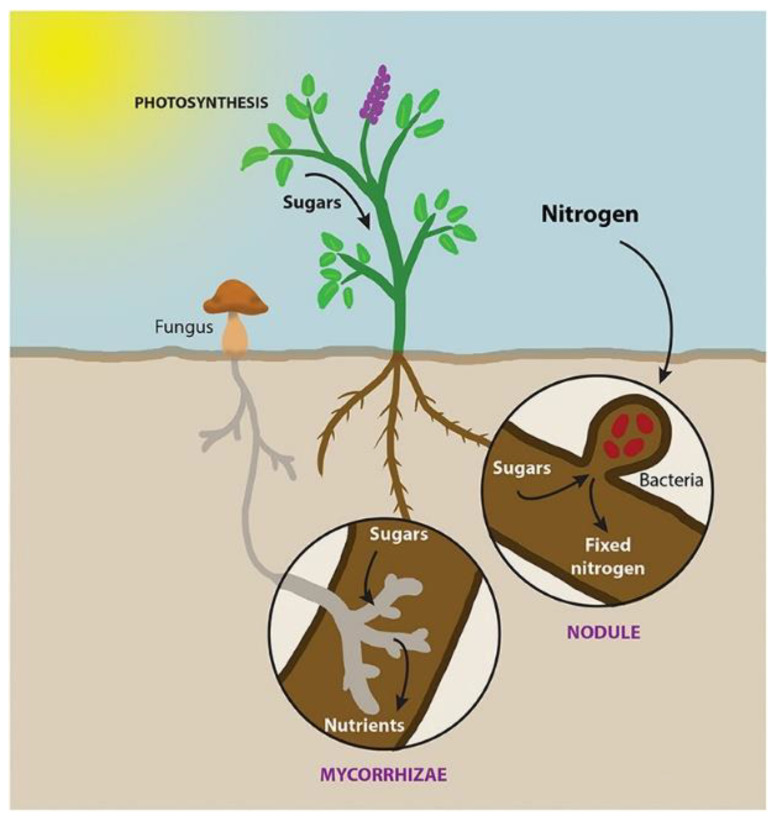
Symbiosis between plant roots and MN.

In Figure 4 we can see that through the MN resources such as carbon (CO_2_) from plants to fungi and water, phosphorus, nitrogen and other nutrients from fungi to plants are exchanged, in addition to an exchange of information through chemical signals when the habitat feels threatened by fire, floods, pests, or predators. It should be noted that this exchange of resources can be between plants of the same species or of different species. Figure 5 shows the symbiosis between plants and the fungal network and how the carbon in the form of sugars flows from the plants to the MN and how the MN fixes the nutrients in the roots of the plants.

The Nobel optimization algorithm DMOA is inspired by the nature of the Mycorrhiza Network (MN) and plant roots with this intimate interaction between these two organisms (plant roots and the network of MN fungi), a symbiosis is generated and it has been discovered that in this relationship [56,57,58,59,60]:

1. There is a communication between plants, which may or may not be of the same species, through a network of fungi (MN).
2. There is an exchange of resources between plants through the fungal network (MN).
3. There is a defensive behavior against predators that can be insects or animals, for the survival of the whole habitat (plants and fungi).
4. The colonization of a forest through a fungal network (MN) thrives much more than a forest where there is no exchange of information and resources.

The launch and publication of the DMOA algorithm has just been carried out in 2022 [61].

**Figure 6:**
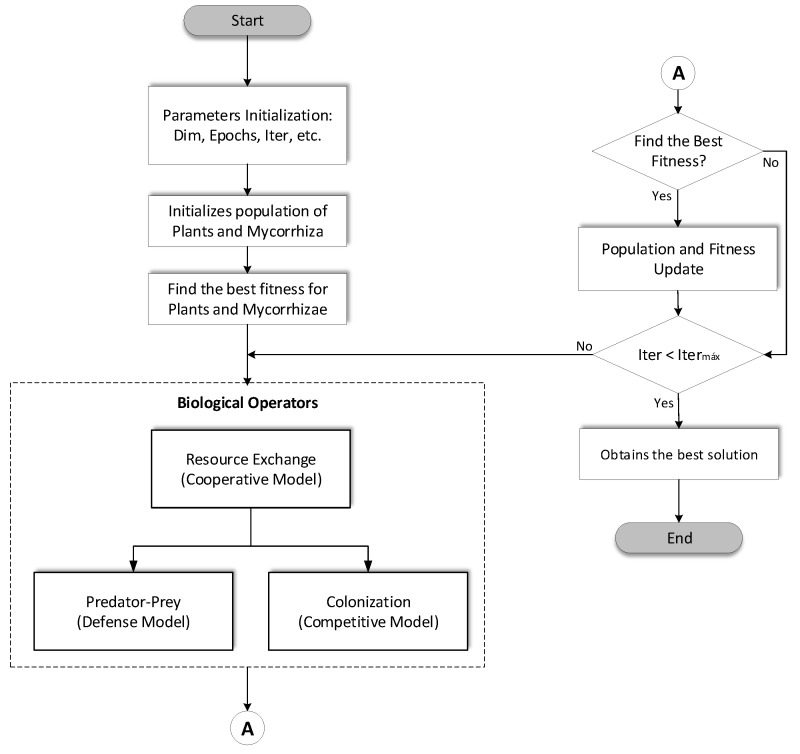
DMOA Flowchart.

Figure 6 describes the flowchart of the DMOA algorithm: we initialize the parameters such as dimensions, epochs, number of iterations, etc., and we also initialize the two populations of plants and mycorrhizae; with these populations we find the best fitness of plants and mycorrhizae, while with these results we use the biological operators. The first operator is represented by the Lotka-Volterra System of Discrete Equations (LVSDE) Cooperative Model [62], whose result has inference on the other two models represented by LVSDE, Defense and Competitive [63,64], and in this frequency we evaluate the fitness to determine if it is better than the previous one and we update the same as the populations, if not we continue with the next iteration and continue the calculation with the biological operators. If the stop condition is fulfilled we obtain the last solution before evaluation and the algorithm ends.

## 4. Proposed Method

**Figure 7:**
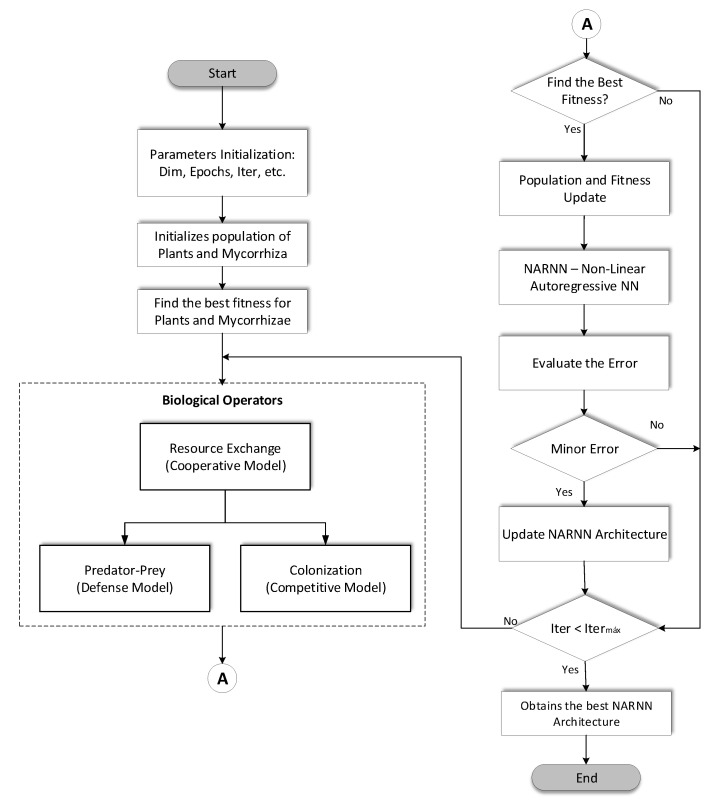
DMOA-NARNN Flowchart.

The proposed method is to use the Discrete Mycorrhiza Optimization Algorithm (DMOA) to optimize the architecture of the Nonlinear Autoregressive Neural Network (NARNN), and as input data we use the Mackey-Glass chaotic time series. In Figure 7 and Algorithm 1 we can find the DMOA-NARNN flowchart and DMOA-NARNN pseudocode, respectively. The DMOA algorithm is explained in Figure 6 in the previous section, in this flowchart we include the optimization of the NARNN, evaluating its results by means of the RMSE, until we manage to find the minimum error of that architecture through the iterations and the populations of the DMOA algorithm (Algorithm 1).
**Algorithm 1 DMOA-NARNN Pseudocode. Discrete Mycorrhiza Optimization Algorithm (DMOA)**  *Objective* min or max f(x), x = (x_1_, x_2_, …, x_d_)  *Define* *parameters* *(a, b, c, d, e, f, x, y)*  *Initialize a population of n plants and mycorrhiza with random solutions*  *Find the best solution **fit** in the initial population*  **while** *(t < maxIter)*      **for** *i* = 1:*n (for n plants and Mycorrhiza population)*      $X_{p}=abs\left(FitA\right)$      $X_{m}=abs\left(FitB\right)$  **end for**  $a=minorX_{p}$  $d=minorX_{m}$  *Apply (LV-Cooperative Model)*  $x_{i}^{t+1}=\frac{\left(ax_{i}-bx_{i}y_{i}\right)}{\left(1-gx_{i}\right)}$  $y_{i}^{t+1}=\frac{\left(dy_{i}+ex_{i}y_{i}\right)}{\left(1+hy_{i}\right)}$  **if** $x_{i}<y_{i}$    $x^{t}=x_{i}$  **else**    $x^{t}=y_{i}$  **end if**  *rand ([1 2])*   **if** *(rand = 1)*     *Apply (LV-Predator-Prey Model)*     $x_{i}^{t+1}=ax_{i}\left(1-x_{i}\right)-bx_{i}y_{i}$     $y_{i}^{t+1}=dx_{i}y_{i}-gy_{i}$  **else**  *   Apply (LV- Competitive Model)*     $x_{i}^{t+1}=\frac{\left(ax_{i}-bx_{i}y_{i}\right)}{\left(1+gx_{i}\right)}$     $y_{i}^{t+1}=\frac{\left(dy_{i}-ex_{i}y_{i}\right)}{\left(1+hy_{i}\right)}$**  end if**  *Evaluate new solutions.*  ***NARNN**-Architecture*  *Evaluate Error*  *Error minor?*  *Update NARNN-Architecture.*  *Find the current best NARNN-Architecture solution.* **end while**

Difference equations often describe the evolution of a particular phenomenon over time. For example, if a given population has discrete generations, the size of *(n +* 1*)* 1st generation *x(n +* 1*)* is a function of the nth generation *x*(*n*). This relationship is expressed by Equation (3):

(3) $$x\left(n+1\right)=f\left(x\left(n\right)\right)$$

We can look at this issue from another perspective. You can generate a sequence from the point *x*_0_, Equation (4):

(4) $$x_{0},\;f\left(x_{0}\right),\;f\left(f\left(x_{0}\right)\right),\;f\left(f\left(f\left(x_{0}\right)\right)\right),\ldots$$

*f(x*_0_*)* is called the first iterate of *x*_0_ under *f*.

Discrete models driven by difference equations are more suitable than continuous models when reproductive generations last only one breeding season (no overlapping generations) [65,66].

An example would be a population that reproduces seasonally, that is, once a year. If we wanted to determine how the population size changes over many years, we could collect data to estimate the population size at the same time each year (say, shortly after the breeding season ends). We know that between the times at which we estimate population size, some individuals will die and that during the breeding season many new individuals will be born, but we ignore changes in population size from day to day, or week to week, and look only at how population size changes from year to year. Thus, when we build a mathematical model of this population, it is reasonable that the model only predicts the population size for each year shortly after the breeding season. In this case, the underlying variable, time, is represented in the mathematical model as increasing in discrete one-year increments.

The LVSDE Equations (5)–(10), have many uses in applied science. These models were first developed in mathematical biology, after which research spread to other fields [67,68,69,70,71].

Discrete Equations (5) and (6) Cooperative Model (Resource-Exchange), for both species, where parameters *a, b, d, e, g,* and *h* are positive constants, *x_i_* and *y_i_* represent the initial conditions of the population for both species and are positive real numbers [72].

The biological operators are represented by LVSDE, the mathematical description of the Discrete Equations (7) and (8) Defense Model (Predator-Prey), where the parameters a, b, d and g are positive constants, *x_i_* and *y_i_* represent the initial population conditions for both species and are positive real numbers [73,74].

Discrete Equations (9) and (10) Competitive Model (Colonization), for two species, where the parameters *a, b, d, e, g*, and *h* are positive constants, *x_i_* and *y_i_* are the populations for each of the species respectively and are positive real numbers. Each of the parameters of the above equations is described in Table 1, [74].

(5) $$x_{i}^{t+1}=\frac{\left(ax_{i}-bx_{i}y_{i}\right)}{\left(1-gx_{i}\right)}$$

(6) $$y_{i}^{t+1}=\frac{\left(dy_{i}+ex_{i}y_{i}\right)}{\left(1+hy_{i}\right)}$$

(7) $$x_{i}^{t+1}=ax_{i}\left(1-x_{i}\right)-bx_{i}y_{i}$$

(8) $$y_{i}^{t+1}=dx_{i}y_{i}-gy_{i}$$

(9) $$x_{i}^{t+1}=\frac{\left(ax_{i}-bx_{i}y_{i}\right)}{\left(1+gx_{i}\right)}$$

(10) $$y_{i}^{t+1}=\frac{\left(dy_{i}-ex_{i}y_{i}\right)}{\left(1+hy_{i}\right)}$$

Table 1 contains the parameters used in all the experiments performed in this research, both those of the DMOA algorithm and those of the NARNN neural network.

The theory of Differential Equations, as well as that of Equations by Differences, can be found in Youssef N. Raffoul. *Qualitative Theory of Volterra Difference Equations* [75], Sigrun Bodine et al., *Asymptotic Integration of Differential and Difference Equations* [76], Takashi Honda et al., *Operator Theoretic Phenomena of the Markov Operators which are Induced by Stochastic Difference Equations* [77], Ronald E. Mickens, *Difference Equations Theory, Applications and Advanced Topics* [78], and Konrad Kitzing, et al., *A Hilbert Space Approach to Difference Equations* [79].

The metric for measuring error is RMSE (Root Mean Square Error) or root mean square deviation, which is one of the most commonly used measures for evaluating the quality of predictions. It shows how far predictions fall from measured true values using Euclidean distance Equation (11), where n is the number of data points, *y_i_* is the *ith* measurement and *ŷ_i_* is the expected prediction [80,81].

(11) $$RMSE=\sqrt{\overset{n}{\underset{i=1}{\sum }}{\left({\hat{y}}_{i}-y_{i}\right)}^{2}}$$

### Mackey-Glass

Chaotic and random time series are both disordered and unpredictable. In extreme cases, the data are so mixed up that those consecutive values seem unrelated to each other. Such disorder would normally eliminate the ability to predict future values from past data.

The Mackey-Glass chaotic time series Equation (12) is a nonlinear differential equation of time delay, and this equation is widely used in the modeling of natural phenomena to make comparisons between different forecasting techniques and regression models [82,83,84], where *a =* 0.1, *b =* 0.2, and *τ =* 17 are real numbers, *t* is the time, and with this setting the series produces chaotic behavior, and we can compare the forecasting performance of DMOA-NARNN with other models in the literature.

(12) $$\dot{y}\left(t\right)=-by\left(t\right)+\frac{cy\left(t-\tau \right)}{1+y^{10}\left(t-\tau \right)}$$

## 5. Results

This section shows the results of the experiments performed in the research involving the Non-Optimized and Optimized results of the method.

Table 2 presents 10 different non-optimized NARNN architectures using only the Mackey-Glass chaotic time series; in the table the columns are represented by: N—Experiment Number, Experiment Name, S—Sample size, T—Training, V—Validation, P—Prediction, HL—Hidden Layers of the NARNN, E—Number of experiment and RMSE (Root Mean Square Error), while the best architecture of the non-optimized NARNN is found in experiment number 4, with the RMSE of 0.1670.

**Figure 8:**
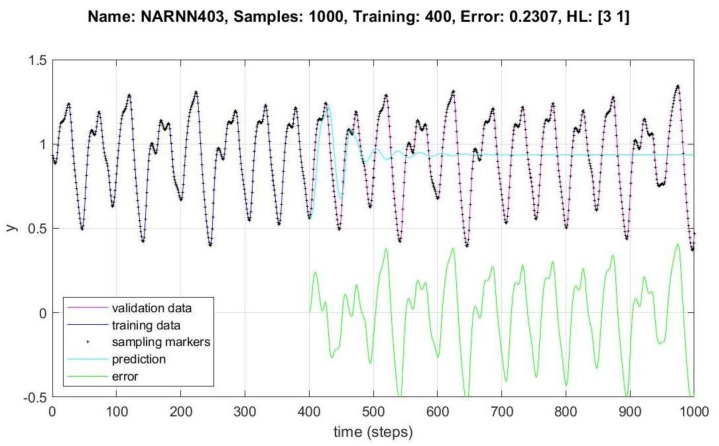
Performance of NARNN403 for 1000 samples.

**Figure 9:**
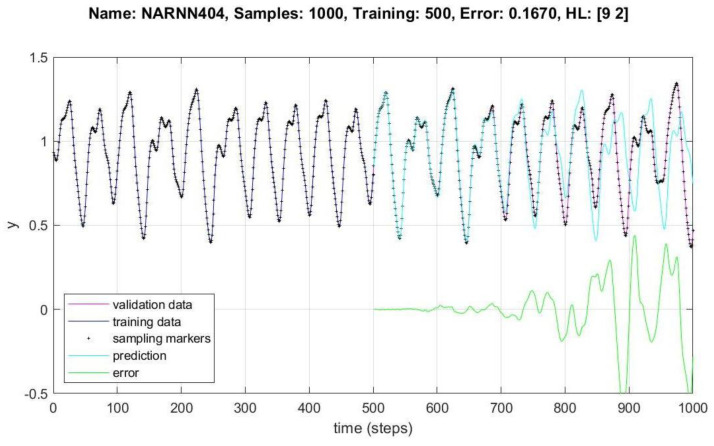
Performance of NARNN404 for 1000 samples.

**Figure 10:**
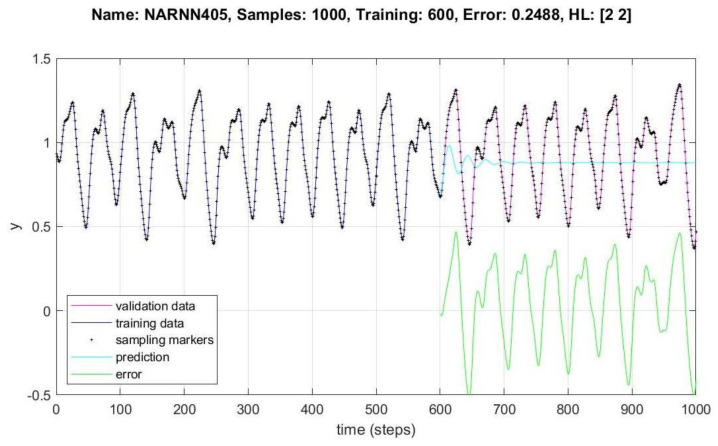
Performance of NARNN405 for 1000 samples.

**Figure 11:**
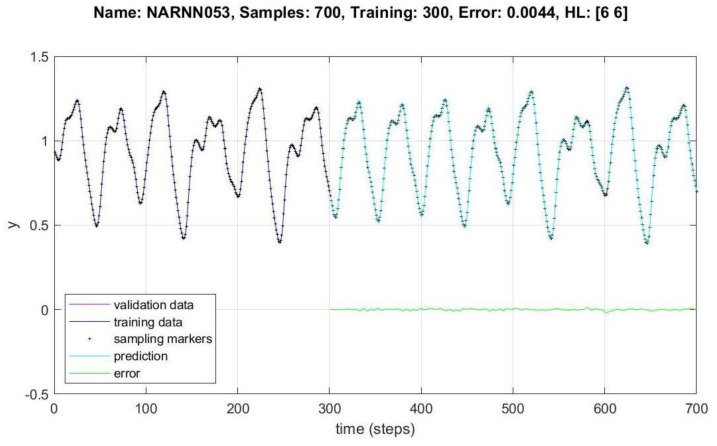
Performance of NARNN053 for 700 samples.

**Figure 12:**
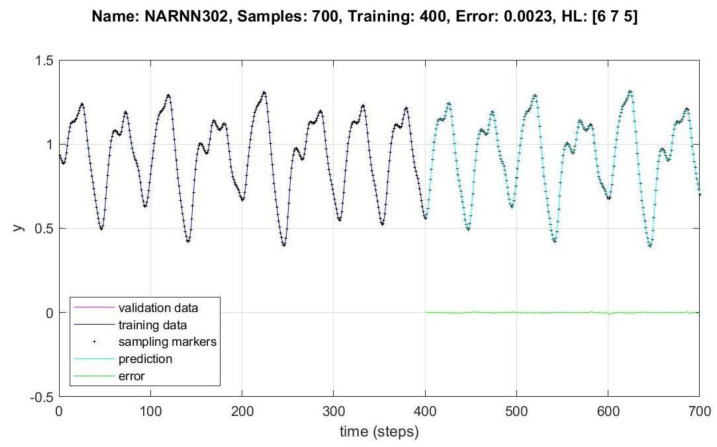
Performance of NARNN302 for 700 samples.

**Figure 13:**
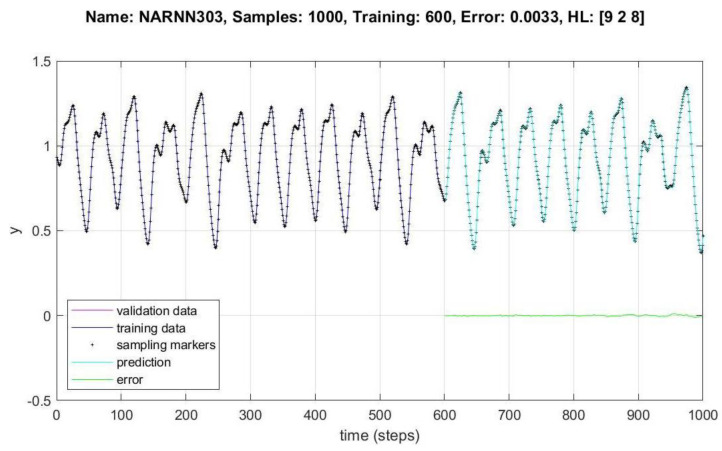
Performance of NARNN303 for 1000 samples.

In Figure 8, Figure 9, Figure 10, Figure 11, Figure 12 and Figure 13, the *y* axes represent the input values (Validation-Training) and output values of the samples (Prediction-Error), the *x* axis represents the number of samples in time, Name is the name of the experiment, Samples is the total number of samples in the experiment, Training is the number of samples for training, Error is the minimum error obtained in the experiment, and *HL* represents the number of neurons in the hidden layers.

Figure 8 shows the behavior of the data for 1000 samples of the NARNN403, obtaining an RMSE of 0.2307, with the reference data at the top of the figure.

Figure 9 and Figure 10 show the data behavior for 1000 samples of the NARNN404 and NARNN405, obtaining an RMSE of 0.167 and 0.2488, respectively, with the reference data at the top of each figure. 

Table 3 shows the results of 39 NARNN architectures optimized with the DMOA algorithm using the Mackey-Glass chaotic time series, in the table the columns are represented by: N - Experiment Number, Experiment Name, S—Sample size, T—Training, V—Validation, P—Prediction, HL—Hidden Layers of the NARNN, I—Number of iterations, Tt—total time of the experiments in seconds, T—time in which the best result was found and RMSE (Root Mean Square Error). The best architecture of the non-optimized NARNN is found in experiment number 31, with the RMSE of 0.0023.

Figure 11, Figure 12 and Figure 13 show the data behavior for 700, 700 and 1000 samples of the NARNN053, NARNN302 and NARNN303, obtaining an RMSE of 0.0044, 0.0023 and 0.0033, respectively, with the reference data at the top of each figure.

As for the complexity of the DMOA algorithm, it is a linear order algorithm that uses the discrete equations of Lotka-Volterra Equations (5)–(10), and in the search to find the global minimum it performs iterations and in each cycle it compares the best previous local minimum with the lowest current minimum and updates the value in the case that this is the case. As for the times, Table 3 shows the times Tt which represents the total time (seconds) of the experiment and T (seconds) the time in which the DMOA algorithm found the lowest local minimum; in terms of its efficiency the algorithm took 1235 s, about 21 min, to find the lowest minimum 0.0023, which seems to us a short time compared to the times used by the method [22] of up to 3 h and a half, the method [21], its experiments took up to 81 h to find the lowest minimum and as for the method [23] it does not provide the times of its experiments.

### 5.1. Statistical Data

Table 4 shows 30 experiments with eight non-optimized NARNNN architectures. Each column represents the total number of samples and the number of training samples used for each architecture (700 × 300), and at the end of the table we can find the results of the total sum, mean and standard deviation for each column.

Table 5 shows 30 experiments with eight optimized NARNNN architectures; each column represents the total number of samples and the number of training samples used by each architecture (700 × 300), and at the end of the table we can find the results of the total sum, mean and standard deviation for each column.

### 5.2. Hypothesis Test

Equation (13) represents Hypothesis Testing, Null Hypothesis Equation (14) and Alternative Hypothesis Equation (15), with which comparisons were made between the non-optimized and optimized experiments of the method proposed here.

(13) $$z=\frac{\left({\bar{x}}_{1}-{\bar{x}}_{2}\right)-D_{0}}{\sqrt{\frac{\sigma_{1}^{2}}{n_{1}}+\frac{\sigma_{2}^{2}}{n_{2}}}}$$

(14) $$H_{0}:\mu_{1}\geq \mu_{2}$$

(15) $$H_{a}:\mu_{1}<\mu_{2}\;claim$$

where ${\bar{x}}_{1}$ is the Mean of sample 1, ${\bar{x}}_{2}$ Mean of sample 2, $\sigma_{1}$ Standard Deviation of sample 1, $\sigma_{2}$ Standard Deviation of sample 2, $n_{1}$ Number of sample data 1, $n_{2}$ Number of sample data 2, $\mu_{1}-\mu_{2}=D_{0}$ and $\mu_{1}-\mu_{2}=D_{0}$.

Significance Level *α =* 0.05, Confidence Level *=* 95%*,* Confidence Level *=* 1 *− α*; 1−0.05 *=* 0.95 o 95%, Since the *p*-value is less than 0.01, the null hypothesis is rejected.

Table 6 and Table 7 show the results of the hypothesis testing done on the non-optimized and optimized methods shown above; of the eight different architectures, the test results show that in only six were the optimized NARNNs better, and the non-optimized NARNNs were better in two.

In Table 6, N and Name represent the number and name of the experiment, respectively. Error is the minimum error found, HL are the Hidden Layers of neural network (1, 2, 3), and N is the number of neurons in each HL. In Table 7, the samples are represented by Total number of samples, T is the training samples, V is the validation samples, P represents the prediction, and *p*-value represents the results of the hypothesis test.

### 5.3. Comparisone with Other Methods

Table 8 shows the comparison with other methods that performed experimentation with the chaotic Mackey-Glass time series, and it can be seen from the table that the lowest error belongs to the optimized NARNN-302.

In Table 8, case number 1, the method is the Optimization of the Fuzzy Integrators in Ensembles of ANFIS Model for Time Series Prediction [21], where the authors use the Mackey-Glass chaotic time series, with genetic optimization of Type-1 Fuzzy Logic System (T1FLS) and Interval Type-2 Fuzzy Logic System (IT2FLS) integrators in Ensemble of ANFIS models and evaluate the results through Root Mean Square Error (RMSE). ANFIS is a hybrid model of a neural network implementation of a TSK (Takagi-Sugeno-Kang) fuzzy inference system. ANFIS applies a hybrid algorithm which integrates BP (Backpropagation) and LSE (least square estimation) algorithms, and thus it has a fast learning speed.

Case number 2 refers to the method using Particle Swarm Optimization of ensemble neural networks with fuzzy aggregation for time series prediction of the Mexican Stock Exchange [22]. In this case, the authors propose an ensemble neural network model with type-2 fuzzy logic for the integration of responses; in addition, the particle swarm optimization method determines the number of modules of the ensemble neural network, the number of layers and number of neurons per layer, and thus the best architecture of the ensemble neural network is obtained. Once this architecture is obtained, the results of the modules with type-1 and type-2 fuzzy logic systems are added, the inputs to the fuzzy system are the responses according to the number of modules of the network, and this is the number of inputs of the fuzzy system.

Case number 3 refers to the Application of Interval Type-2 Fuzzy Neural Networks (IT2FNN) in non-linear identification and time series prediction (MG) [23]. The authors propose IT2FNN models that combine the uncertainty management advantage of type-2 fuzzy sets with the learning capabilities of neural networks. One of the main ideas of this approach is that the proposed IT2FNN architectures can obtain similar or better outputs than type-2 interval fuzzy systems using the Karnik and Mendel (KM) algorithm, but with lower computational cost, which is one of the main disadvantages of KM mentioned in many papers in the literature. Cases 4 and 5 have already been explained earlier in this article.

By making a brief description of the techniques of the different methods above, we can observe the complexity of their designs using optimization algorithms such as PSO and GAs as optimizers, robust Ensemble Neural Networks, T1FLS and IT2FLS, in comparison with our method that uses the optimization algorithm DMOA and NARNNN, which are neural networks with short memory, and according to the results are made precisely for the prediction of time series. In a future work we plan to perform experiments with the RNN LSTM networks, which have short- and long-term memories.

## 6. Discussion of Results

The use of metaheuristics in the optimization of methods is a constant in all research work in artificial intelligence, and in this work the DMOA algorithm was used to optimize the architecture of the NARNN neural network using the MG chaotic series as input data. We also performed experiments without optimizing the NARNN network, while with the optimization we performed experiments with 39 different architectures, and without optimization we performed experiments with 10 different architectures. When we performed the optimization we found an extremely fast algorithm that found the right architecture with very satisfactory results. Of the 39 different optimized architectures, the one that gave us the best results was number 31 (narAll303) Table 3, a NARNN network with three hidden layers of 6, 7, and 5 neurons, respectively. With this architecture we performed 3000 experiments with a total time of 5353 s and in the second 1235 we obtained the best result of 0.0023 (error). Of the 10 experiments without optimization, with architecture number 4 (narAll404) Table 2, a NARNN network with two hidden layers of 9 and 2 neurons, respectively, we also performed 3000 experiments with this architecture and obtained the best result of 0.1670 (error). We performed eight hypothesis tests under equal conditions with these results and found that in five tests the NARNN architectures optimized with the DMOA algorithm were better and in three tests the non-optimized architectures were better, as shown in Table 6. We also performed error comparisons with three other different methods of which the DMOA-NARNN was better, as shown in Table 8.

## 7. Conclusions

A total of 49 different architectures were designed, of which 10 non-optimized and 39 were optimized by the DMOA algorithm, 30,000 experiments were performed with the non-optimized architectures, and approximately 110,000 experiments were performed with the optimized architectures. A total of 700, 1000 and 1500 samples were generated with the MG chaotic time series, of which between 300 and 1000 were used for training, between 300 and 900 were used for validation in different combinations, and between 300 and 900 points were generated as prediction points, as can be seen in Table 2 and Table 3. The design of the NARNN architectures were two and three hidden layers, with neurons in the range of 2–9, and the graphs of the most representative results of the non-optimized and optimized NARNNs are presented in Figure 8, Figure 9, Figure 10, Figure 11, Figure 12 and Figure 13.

The optimization of the NARNN network with the DMOA algorithm obtained good results, better than without optimizing the network, and better than the other methods with which it was compared, although not all of the optimized architectures were better in the hypothesis test (only five of them were), the results of the error were much better, as can be seen in Table 7. In the comparison with other methods, the results were also better, as demonstrated in Table 8. We were also able to verify that the DMOA optimization algorithm is fast and efficient, which was really the reason for this research. We wish to continue investigating the efficiency of the algorithm in the optimization of architectures with other types of neural networks, also in Fuzzy Logic Systems Type-1 and Type-2, and also to do the same with the optimization algorithm CMOA (Continuous Mycorrhiza Optimization Algorithm). In addition, the proposed algorithm can be applied to robots, microsystems, sensors, devices, MEMS, microfluidics, piezoelectricity, motors, biosensors, 3D printing, etc.

We also intend to conduct further research and experimentation with the DMOA method and other time series. We will also consider the DMOA and the LSTM (Long Short-Term Memory) Neural Regression Network for Mackey-Glass time series, weather and financial forecasting, and we are interested in hybridizing the method with Interval Type-2 Fuzzy Logic System (IT2FLS), and Generalized Type-2 Fuzzy Logic System (GT2FLS).

## Figures and Tables

**Table 1:** DMOA-NARNN Parameters.

Parameter	Description	Value
DMOA—Parameters:		
$x_{i}^{t+1}$	Population x at time t	
$y_{i}^{t+1}$	Population y at time t	
$x_{i}$	Grow rates of populations x at time t	
$y_{i}$	Grow rates of populations y at time t	
*t*	time	
*a*	Population growth rate x	0.01
*b*	Influence of population x on itself	0.02
*g*	Influence of population y on population x	0.06
*d*	Population growth rate y	0
*e*	Influence of population x on population y	1.7
*h*	Influence of population y on itself	0.09
*x*	Initial population in x	0.0002
*y*	Initial population in y	0.0006
In the absence of population *x =* 0, In the absence of population *y =* 0		
*a, b, c, d, e* and *f*—are positive constants		
Population	Population size	20
Populations	Number of populations	2
Dimensions	Dimensions size	30, 50, 100
Epochs	Number of epochs	30
Iterations	Iteration’s size	30, 50, 100, 500
NARNN—Parameters:		
h	Hidden Layers	2, 3
n	Neurons	2–10
	Vector time delay	01:06:19

**Table 2:** Results of non-optimized experiments.

N	Name	S	T	V	P	HL	E	RMSE
1	NARNN401	700	300	400	400	[1 1]	3000	0.2777
2	NARNN402	700	400	300	300	[1 1]	3000	0.1683
3	NARNN403	1000	400	600	600	[3 1]	3000	0.2307
4	NARNN404	1000	500	500	500	[9 2]	3000	0.1670
5	NARNN405	1000	600	400	400	[2 2]	3000	0.2488
6	NARNN406	1500	600	900	900	[4 1 9]	3000	0.2550
7	NARNN407	1500	700	800	800	[8 5 2]	3000	0.2158
8	NARNN408	1500	800	700	700	[1 2 1]	3000	0.4001
9	NARNN409	1500	900	600	600	[2 3 2]	3000	0.2810
10	NARNN410	1500	1000	500	500	[6 6 8]	3000	0.1712

**Table 3:** Results of optimized experiments.

N	Name	S	T	V	P	HL	I	Tt	T	RMSE
1	NARNN041	700	300	400	400	[5 6]	900			0.0114
2	NARNN042	700	300	400	400	[5 6]	1000			0.0054
3	NARNN043	700	300	400	400	[5 6]	1000			0.1012
4	NARNN053	700	300	400	400	[6 6]	500		405	0.0044
5	NARNN055	700	300	400	400	[6 1]	100		4	0.0202
6	NARNN056	700	300	400	400	[6 6]	500		486	0.0067
7	NARNN057	700	300	400	400	[4 3]	1000		807	0.0075
8	NARNN058	700	300	400	400	[8 7]	2500		154	0.0131
9	NARNN058r	700	300	400	400	[6 5]	2500		75	0.0202
10	NARNN059	700	300	400	400	[5 4]	1000		445	0.0061
11	NARNN060	700	300	400	400	[6 2]	1000		259	0.0081
12	NARNN061	700	300	400	400	[5 6]	1000		496	0.0044
13	NARNN062	700	400	300	300	[5 6]	5000		688	0.0024
14	NARNN201	700	400	300	300	[9 4]	5000	2948	1476	0.0024
15	NARNN202	1000	600	400	400	[7 5]	5000		1905	0.0035
16	NARNN203	1000	500	500	500	[4 7]	1000		681	0.0084
17	NARNN204	1000	400	600	600	[8 3]	1000		917	0.0144
18	NARNN205	1000	400	600	600	[7 8]	5000	2849	2741	0.0076
19	NARNN206	1000	500	500	500	[8 5]	5000	3262	2517	0.0059
20	NARNN207	1000	600	400	400	[6 2]	5000	3659	1822	0.0047
21	NARNN208	1500	600	900	900	[7 9]	5000	3666	1686	0.0187
22	NARNN209	1500	700	800	800	[7 7]	5000	4039	2665	0.0104
23	NARNN210p	1500	800	700	700	[7 3]	6000	5750	5226	0.0122
24	NARNN211	1500	900	600	600	[7 3]	3000	5439	1658	0.0136
25	NARNN212	1500	900	600	600	[5 6]	3000	6479	606	0.0055
26	NARNN213	1500	800	700	700	[5 6]	3000	12685	470	0.0080
27	NARNN214p	1500	700	800	800	[5 6]	5000	6183	4739	0.0157
28	NARNN215	1500	600	900	900	[5 6]	3000	2848	1910	0.0230
29	NARNN311	1500	1000	500	500	[6 1]	3000	6919	1917	0.0047
30	NARNN301	700	300	400	400	[7 8 3]	2000	1371	1313	0.0052
31	NARNN302	700	400	300	300	[6 7 5]	3000	5353	1235	0.0023
32	NARNN303	1000	600	400	400	[9 2 8]	3000	2348	2003	0.0033
33	NARNN304	1000	500	500	500	[4 5 4]	3000	5072	1334	0.0040
34	NARNN305	1000	400	600	600	[8 7 1]	3000	4435	2414	0.0070
35	NARNN306	1500	600	900	900	[5 5 1]	3000	5610	2309	0.0851
36	NARNN307	1500	700	800	800	[8 4 1]	3000	3151	1664	0.0245
37	NARNN308	1500	800	700	700	[8 1 1]	3000	3161	76	0.0127
38	NARNN309	1500	900	600	600	[8 2 7]	3000	3175	1502	0.0098
39	NARNN310	1500	1000	500	500	[8 3 8]	3000	3333	1050	0.0074

**Table 4:** 30 Experiments with non-optimized NARNN.

No	Non Optimized							
	2 Hidden Layers					3 Hidden Layers		
	700 × 300	700 × 400	1000 × 400	1000 × 500	1000 × 600	1500 × 600	1500 × 700	1500 × 800
1	3.58 × 10^−1^	3.17 × 10^−1^	2.84 × 10^−1^	2.45 × 10^−1^	3.01 × 10^−1^	9.88 × 10^−1^	2.78 × 10^−1^	7.27 × 10^−1^
2	2.97 × 10^−1^	2.39 × 10^−1^	2.81 × 10^−1^	2.32 × 10^−1^	2.96 × 10^−1^	3.41 × 10^−1^	2.72 × 10^−1^	5.39 × 10^−1^
3	2.84 × 10^−1^	2.35 × 10^−1^	2.77 × 10^−1^	2.26 × 10^−1^	2.90 × 10^−1^	3.39 × 10^−1^	2.44 × 10^−1^	4.67 × 10^−1^
4	2.81 × 10^−1^	2.23 × 10^−1^	2.66 × 10^−1^	2.25 × 10^−1^	2.55 × 10^−1^	3.38 × 10^−1^	2.36 × 10^−1^	4.35 × 10^−1^
5	2.68 × 10^−1^	2.22 × 10^−1^	2.60 × 10^−1^	2.22 × 10^−1^	2.49 × 10^−1^	3.27 × 10^−1^	2.34 × 10^−1^	4.27 × 10^−1^
6	2.66 × 10^−1^	2.22 × 10^−1^	2.47 × 10^−1^	2.06 × 10^−1^	2.47 × 10^−1^	3.19 × 10^−1^	2.33 × 10^−1^	4.23 × 10^−1^
7	2.65 × 10^−1^	2.21 × 10^−1^	2.39 × 10^−1^	2.03 × 10^−1^	2.44 × 10^−1^	3.16 × 10^−1^	2.31 × 10^−1^	4.18 × 10^−1^
8	2.62 × 10^−1^	2.19 × 10^−1^	2.39 × 10^−1^	1.83 × 10^−1^	2.44 × 10^−1^	3.07 × 10^−1^	2.30 × 10^−1^	4.18 × 10^−1^
9	2.62 × 10^−1^	2.16 × 10^−1^	2.34 × 10^−1^	1.75 × 10^−1^	2.43 × 10^−1^	3.00 × 10^−1^	2.30 × 10^−1^	4.08 × 10^−1^
10	2.52 × 10^−1^	2.13 × 10^−1^	2.33 × 10^−1^	1.74 × 10^−1^	2.40 × 10^−1^	2.92 × 10^−1^	2.27 × 10^−1^	4.02 × 10^−1^
11	2.51 × 10^−1^	2.12 × 10^−1^	2.32 × 10^−1^	1.73 × 10^−1^	2.36 × 10^−1^	2.73 × 10^−1^	2.27 × 10^−1^	3.98 × 10^−1^
12	2.50 × 10^−1^	2.11 × 10^−1^	2.31 × 10^−1^	1.72 × 10^−1^	2.34 × 10^−1^	2.73 × 10^−1^	2.21 × 10^−1^	3.94 × 10^−1^
13	2.50 × 10^−1^	2.10 × 10^−1^	2.31 × 10^−1^	1.71 × 10^−1^	2.33 × 10^−1^	2.69 × 10^−1^	2.19 × 10^−1^	3.91 × 10^−1^
14	2.49 × 10^−1^	2.09 × 10^−1^	2.29 × 10^−1^	1.69 × 10^−1^	2.31 × 10^−1^	2.63 × 10^−1^	2.16 × 10^−1^	3.89 × 10^−1^
15	2.49 × 10^−1^	2.08 × 10^−1^	2.29 × 10^−1^	1.69 × 10^−1^	2.15 × 10^−1^	2.60 × 10^−1^	2.14 × 10^−1^	2.53 × 10^−1^
16	2.43 × 10^−1^	2.07 × 10^−1^	2.29 × 10^−1^	1.68 × 10^−1^	2.11 × 10^−1^	2.47 × 10^−1^	2.04 × 10^−1^	2.51 × 10^−1^
17	2.43 × 10^−1^	2.00 × 10^−1^	2.28 × 10^−1^	1.67 × 10^−1^	2.10 × 10^−1^	2.33 × 10^−1^	1.97 × 10^−1^	2.42 × 10^−1^
18	2.43 × 10^−1^	1.95 × 10^−1^	2.27 × 10^−1^	1.66 × 10^−1^	2.08 × 10^−1^	2.29 × 10^−1^	1.95 × 10^−1^	2.38 × 10^−1^
19	2.36 × 10^−1^	1.93 × 10^−1^	2.23 × 10^−1^	1.66 × 10^−1^	2.07 × 10^−1^	2.29 × 10^−1^	1.87 × 10^−1^	2.33 × 10^−1^
20	2.36 × 10^−1^	1.89 × 10^−1^	2.17 × 10^−1^	1.66 × 10^−1^	2.05 × 10^−1^	2.27 × 10^−1^	1.86 × 10^−1^	2.27 × 10^−1^
21	2.36 × 10^−1^	1.89 × 10^−1^	2.13 × 10^−1^	1.54 × 10^−1^	2.02 × 10^−1^	2.12 × 10^−1^	1.86 × 10^−1^	2.25 × 10^−1^
22	2.33 × 10^−1^	1.89 × 10^−1^	2.01 × 10^−1^	1.52 × 10^−1^	2.02 × 10^−1^	2.06 × 10^−1^	1.82 × 10^−1^	2.25 × 10^−1^
23	2.33 × 10^−1^	1.88 × 10^−1^	1.95 × 10^−1^	1.35 × 10^−1^	2.02 × 10^−1^	2.04 × 10^−1^	1.75 × 10^−1^	2.24 × 10^−1^
24	2.28 × 10^−1^	1.82 × 10^−1^	1.90 × 10^−1^	1.30 × 10^−1^	2.01 × 10^−1^	2.03 × 10^−1^	1.72 × 10^−1^	2.23 × 10^−1^
25	2.26 × 10^−1^	1.78 × 10^−1^	1.85 × 10^−1^	1.19 × 10^−1^	2.00 × 10^−1^	1.98 × 10^−1^	1.69 × 10^−1^	2.22 × 10^−1^
26	2.25 × 10^−1^	1.78 × 10^−1^	1.85 × 10^−1^	1.19 × 10^−1^	1.97 × 10^−1^	1.96 × 10^−1^	1.67 × 10^−1^	2.19 × 10^−1^
27	2.24 × 10^−1^	1.77 × 10^−1^	1.74 × 10^−1^	1.14 × 10^−1^	1.74 × 10^−1^	1.96 × 10^−1^	1.59 × 10^−1^	2.16 × 10^−1^
28	2.23 × 10^−1^	1.70 × 10^−1^	1.60 × 10^−1^	1.09 × 10^−1^	1.62 × 10^−1^	1.95 × 10^−1^	1.58 × 10^−1^	2.15 × 10^−1^
29	2.10 × 10^−1^	1.70 × 10^−1^	1.45 × 10^−1^	1.01 × 10^−1^	1.32 × 10^−1^	1.92 × 10^−1^	1.57 × 10^−1^	2.14 × 10^−1^
30	1.96 × 10^−1^	1.65 × 10^−1^	5.20 × 10^−2^	6.12 × 10^−2^	7.29 × 10^−2^	1.61 × 10^−1^	1.50 × 10^−1^	1.94 × 10^−1^
Sum:	7.48 × 10^+0^	6.15 × 10^+0^	6.53 × 10^+0^	4.97 × 10^+0^	6.54 × 10^+0^	8.34 × 10^+0^	6.16 × 10^+0^	9.86 × 10^+0^
Mean:	2.49 × 10^−1^	2.05 × 10^−1^	2.18 × 10^−1^	1.66 × 10^−1^	2.18 × 10^−1^	2.78 × 10^−1^	2.05 × 10^−1^	3.29 × 10^−1^
SD:	3.00 × 10^−2^	2.91 × 10^−2^	4.59 × 10^−2^	4.25 × 10^−2^	4.58 × 10^−2^	1.44 × 10^−1^	3.40 × 10^−2^	1.27 × 10^−1^

**Table 5:** 30 Experiments with optimized NARNN.

No	Optimized							
	2 Hidden Layers					3 Hidden Layers		
	700 × 300	700 × 400	1000 × 400	1000 × 500	1000 × 600	1500 × 600	1500 × 700	1500 × 800
1	1.72 × 10^−1^	4.62 × 10^−2^	2.63 × 10^−1^	1.97 × 10^−1^	2.71 × 10^−1^	3.88 × 10^−1^	2.85 × 10^−1^	2.93 × 10^−1^
2	1.52 × 10^−1^	4.44 × 10^−2^	2.34 × 10^−1^	1.97 × 10^−1^	1.92 × 10^−1^	3.07 × 10^−1^	2.67 × 10^−1^	2.67 × 10^−1^
3	1.35 × 10^−1^	4.21 × 10^−2^	2.32 × 10^−1^	1.92 × 10^−1^	1.92 × 10^−1^	3.06 × 10^−1^	2.53 × 10^−1^	2.66 × 10^−1^
4	1.34 × 10^−1^	4.07 × 10^−2^	2.31 × 10^−1^	1.91 × 10^−1^	1.91 × 10^−1^	2.97 × 10^−1^	2.43 × 10^−1^	2.36 × 10^−1^
5	1.18 × 10^−1^	3.98 × 10^−2^	2.30 × 10^−1^	1.87 × 10^−1^	1.91 × 10^−1^	2.94 × 10^−1^	2.39 × 10^−1^	2.29 × 10^−1^
6	1.09 × 10^−1^	3.73 × 10^−2^	2.28 × 10^−1^	1.86 × 10^−1^	1.89 × 10^−1^	2.90 × 10^−1^	2.36 × 10^−1^	2.28 × 10^−1^
7	1.07 × 10^−1^	3.59 × 10^−2^	2.27 × 10^−1^	1.86 × 10^−1^	1.89 × 10^−1^	2.86 × 10^−1^	2.25 × 10^−1^	2.27 × 10^−1^
8	9.68 × 10^−2^	3.40 × 10^−2^	2.26 × 10^−1^	1.83 × 10^−1^	1.89 × 10^−1^	2.74 × 10^−1^	2.22 × 10^−1^	2.24 × 10^−1^
9	9.67 × 10^−2^	3.26 × 10^−2^	2.22 × 10^−1^	1.80 × 10^−1^	1.86 × 10^−1^	2.74 × 10^−1^	2.21 × 10^−1^	2.23 × 10^−1^
10	9.61 × 10^−2^	3.08 × 10^−2^	2.20 × 10^−1^	1.75 × 10^−1^	1.83 × 10^−1^	2.72 × 10^−1^	2.19 × 10^−1^	2.17 × 10^−1^
11	9.54 × 10^−2^	3.06 × 10^−2^	2.18 × 10^−1^	1.74 × 10^−1^	1.74 × 10^−1^	2.70 × 10^−1^	2.17 × 10^−1^	2.13 × 10^−1^
12	9.23 × 10^−2^	2.19 × 10^−2^	2.18 × 10^−1^	1.73 × 10^−1^	1.69 × 10^−1^	2.66 × 10^−1^	2.16 × 10^−1^	2.09 × 10^−1^
13	9.01 × 10^−2^	2.18 × 10^−2^	2.16 × 10^−1^	1.72 × 10^−1^	1.69 × 10^−1^	2.62 × 10^−1^	2.14 × 10^−1^	2.08 × 10^−1^
14	8.57 × 10^−2^	2.14 × 10^−2^	2.12 × 10^−1^	1.72 × 10^−1^	1.57 × 10^−1^	2.53 × 10^−1^	2.04 × 10^−1^	2.07 × 10^−1^
15	8.11 × 10^−2^	1.91 × 10^−2^	2.09 × 10^−1^	1.72 × 10^−1^	1.50 × 10^−1^	2.53 × 10^−1^	1.78 × 10^−1^	2.05 × 10^−1^
16	7.47 × 10^−2^	1.91 × 10^−2^	2.08 × 10^−1^	1.71 × 10^−1^	1.40 × 10^−1^	2.51 × 10^−1^	1.74 × 10^−1^	2.02 × 10^−1^
17	7.13 × 10^−2^	1.79 × 10^−2^	1.89 × 10^−1^	1.69 × 10^−1^	1.35 × 10^−1^	2.44 × 10^−1^	1.73 × 10^−1^	1.99 × 10^−1^
18	6.11 × 10^−2^	1.77 × 10^−2^	1.89 × 10^−1^	1.69 × 10^−1^	1.33 × 10^−1^	2.37 × 10^−1^	1.71 × 10^−1^	1.98 × 10^−1^
19	5.99 × 10^−2^	1.31 × 10^−2^	1.85 × 10^−1^	1.67 × 10^−1^	1.32 × 10^−1^	2.33 × 10^−1^	1.58 × 10^−1^	1.98 × 10^−1^
20	5.76 × 10^−2^	1.25 × 10^−2^	1.78 × 10^−1^	1.66 × 10^−1^	1.32 × 10^−1^	2.28 × 10^−1^	1.53 × 10^−1^	1.90 × 10^−1^
21	5.43 × 10^−2^	1.12 × 10^−2^	1.78 × 10^−1^	1.66 × 10^−1^	1.29 × 10^−1^	2.27 × 10^−1^	1.53 × 10^−1^	1.78 × 10^−1^
22	5.35 × 10^−2^	1.02 × 10^−2^	1.77 × 10^−1^	1.64 × 10^−1^	1.29 × 10^−1^	2.24 × 10^−1^	1.52 × 10^−1^	1.66 × 10^−1^
23	3.96 × 10^−2^	9.31 × 10^−3^	1.76 × 10^−1^	1.45 × 10^−1^	1.28 × 10^−1^	2.21 × 10^−1^	1.50 × 10^−1^	1.65 × 10^−1^
24	3.58 × 10^−2^	9.11 × 10^−3^	1.74 × 10^−1^	1.42 × 10^−1^	1.28 × 10^−1^	2.12 × 10^−1^	1.45 × 10^−1^	1.61 × 10^−1^
25	2.82 × 10^−2^	6.99 × 10^−3^	1.71 × 10^−1^	1.37 × 10^−1^	1.27 × 10^−1^	1.98 × 10^−1^	1.44 × 10^−1^	1.58 × 10^−1^
26	2.27 × 10^−2^	5.98 × 10^−3^	1.71 × 10^−1^	1.30 × 10^−1^	1.26 × 10^−1^	1.98 × 10^−1^	1.42 × 10^−1^	1.58 × 10^−1^
27	2.13 × 10^−2^	5.63 × 10^−3^	1.54 × 10^−1^	1.16 × 10^−1^	1.26 × 10^−1^	1.92 × 10^−1^	1.41 × 10^−1^	9.36 × 10^−2^
28	1.89 × 10^−2^	4.96 × 10^−3^	1.19 × 10^−1^	1.15 × 10^−1^	1.24 × 10^−1^	1.91 × 10^−1^	1.38 × 10^−1^	6.64 × 10^−2^
29	1.84 × 10^−2^	4.21 × 10^−3^	1.03 × 10^−1^	8.25 × 10^−2^	1.23 × 10^−1^	1.88 × 10^−1^	1.37 × 10^−1^	5.36 × 10^−2^
30	1.14 × 10^−2^	3.52 × 10^−3^	5.66 × 10^−2^	1.51 × 10^−2^	1.11 × 10^−1^	1.75 × 10^−1^	1.35 × 10^−1^	4.84 × 10^−2^
Sum:	2.29 × 10^+0^	6.50 × 10^−1^	5.81 × 10^+0^	4.79 × 10^+0^	4.71 × 10^+0^	7.51 × 10^+0^	5.70 × 10^+0^	5.69 × 10^+0^
Mean:	7.63 × 10^−2^	2.17 × 10^−2^	1.94 × 10^−1^	1.60 × 10^−1^	1.57 × 10^−1^	2.50 × 10^−1^	1.90 × 10^−1^	1.90 × 10^−1^
SD:	4.21 × 10^−2^	1.38 × 10^−2^	4.35 × 10^−2^	3.81 × 10^−2^	3.52 × 10^−2^	4.60 × 10^−2^	4.47 × 10^−2^	5.91 × 10^−2^

**Table 6:** Data for the eight non-optimized and optimized architectures.

NARNN—Non-OPTIMIZED						NARNN—OPTIMIZED				
N	Name	Error	HL and N			Name	Error	HL and N		
			1	2	3			1	2	3
1	NARNN401	0.2777	1	1		NARNN053	0.0052	6	6	
2	NARNN402	0.1683	1	1		NARNN062	0.0023	5	6	
3	NARNN403	0.2307	3	1		NARNN205	0.0070	7	8	
4	NARNN404	0.1670	9	2		NARNN206	0.0040	8	5	
5	NARNN405	0.2488	2	2		NARNN207	0.0033	6	2	
6	NARNN406	0.2550	4	1	9	NARNN306	0.0851	5	5	1
7	NARNN407	0.2158	8	5	2	NARNN307	0.0245	8	4	1
8	NARNN408	0.4001	1	2	1	NARNN308	0.0127	8	1	1

**Table 7:** Hypothesis test results of the eight non-optimized and optimized NARNNs.

Samples				Results	
				Non-Optimized	Optimized
Total	T	V	P	*p*-Value	
700	300	400	400		8.25 × 10^−26^
700	400	300	300		6.00 × 10^−38^
1000	400	600	600		4.23 × 10^−2^
1000	500	500	500	5.59 × 10^−1^	
1000	600	400	400		3.13 × 10^−7^
1500	600	900	900	3.25 × 10^−1^	
1500	700	800	800	1.46 × 10^−1^	
1500	800	700	700		1.14 × 10^−6^

**Table 8:** Error comparison with three different methods and the non-optimized and optimized NARNN.

N	Experiment Description						Error	Serie	Ref
1	Genetic Algorithm—Ensemble ANFIS—T1FLS—IT2FLS						0.0219	MG	[21]
2	Ensemble Neural Network Architecture						0.008945	MSE	[22]
	2 Modules, 2 Hidden Layers, 2116 and 2128 Neurons respectively, PSO Optimized								
3	SNR(dB)	ANFIS	IT2FNN-0	IT2FNN-1	IT2FNN-2	IT2FNN-3	0.0028	MG	[23]
	30	0.0225	0.0106	0.0079	0.0045	0.0028			
4	NARNN-DMOA (No-Optimized)						0.167	MG	
5	NARNN-DMOA (Optimized)						0.0023	MG	

## Data Availability

Not applicable.

## References

[1] Diwekar U.M. (2020). Introduction to Applied Optimization.

[2] Ghaemi M.B., Gharakhanlu N., Rassias T.M., Saadati R. (2021). Advances in Matrix Inequalities.

[3] Lange K. (2013). Optimization Second Edition.

[4] Kochenderfer M.J., Wheeler T.A. (2019). Algorithms for Optimization.

[5] Adam S.P., Alexandropoulos S.N., Pardalos P.M., Vrahatis M.N., Demetriou I., Pardalos P. (2019). No Free Lunch Theorem: A Review. Approximation and Optimization.

[6] Bianchi F.M., Maiorino E., Kampffmeyer M.C., Rizzi A., Jenssen R. (2017). An overview and comparative analysis of Recurrent Neural Networks for Short Term Load Forecasting. arXiv.

[7] Schäfer A.M., Zimmermann H.G., Kollias S.D., Stafylopatis A., Duch W., Oja E. (2006). Recurrent Neural Networks Are Universal Approximators.

[8] Brownlee J. (2019). Deep Learning for Time Series Forecasting Predict the Future with MLPs, CNNs and LSTMs in Python.

[9] Graves A. (2012). Sequence transduction with recurrent neural networks. arXiv.

[10] Graves A. (2013). Generating sequences with recurrent neural networks. arXiv.

[11] Pascanu R., Mikolov T., Bengio Y. On the difficulty of training Recurrent Neural Networks. Proceedings of the 30th International Conference on Machine Learning, ICML 2013, JMLR.org.

[12] Mikolov T. (2012). Statistical Language Models Based on Neural Networks. Ph.D. Thesis.

[13] Sutskever I., Martens J., Hinton G. Generating Text with Recurrent Neural Networks. Proceedings of the 28th International Conference on Machine Learning. ICML 2011.

[14] Graves A. (2011). Practical variational inference for neural networks. Advances in Neural Information Processing Systems.

[15] Mikolov T., Sutskever I., Chen K., Corrado G.S., Dean J. (2013). Distributed representations of words and phrases and their compositionality. Advances in Neural Information Processing Systems.

[16] Oord A., Dieleman S., Zen H., Vinyals K.S.O., Graves A., Kalchbrenner N., Senior A., Kavukcuoglu K. (2016). A generative model for raw audio. arXiv.

[17] Graves A., Schmidhuber J. (2009). Offline handwriting recognition with multidimensional recurrent neural networks. Advances in Neural Information Processing Systems.

[18] Graves A., Fernández S., Liwicki M., Bunke H., Schmidhuber J. Unconstrained On-line Handwriting Recognition with Recurrent Neural Networks. Proceedings of the Advances in Neural Information Processing Systems.

[19] Gregor K., Danihelka I., Graves A., Rezende D., Wierstra D. DRAW: A recurrent neural network for image generation. Proceedings of the 32nd International Conference on Machine Learning, PMLR.

[20] Hochreiter S., Schmidhuber J. (1997). Long short-term memory. Neural Comput..

[21] Soto J., Melin P. Optimization of the Fuzzy Integrators in Ensembles of ANFIS Model for Time Series Prediction: The case of Mackey-Glass. Proceedings of the 2015 Conference of the International Fuzzy Systems Association and the European Society for Fuzzy Logic and Technology (IFSA-EUSFLAT-15).

[22] Pulido M., Melin P., Castillo O. (2014). Particle swarm optimization of ensemble neural networks with fuzzy aggregation for time series prediction of the Mexican Stock Exchange. Inf. Sci..

[23] Castillo O., Castro J.R., Melin P., Rodríguez-Díaz A. (2013). Application of interval type-2 fuzzy neural networks in non-linear identification and time series prediction. Soft Comput..

[24] Amador-Angulo L., Castillo O. (2015). Amador-Angulo, L.; Castillo, O. A Fuzzy Bee Colony Optimization Algorithm Using an Interval Type-2 Fuzzy Logic System for Trajectory Control of a Mobile Robot. Mexican International Conference on Artificial Intelligence.

[25] Zangeneh M., Aghajari E., Forouzanfar M. (2020). A Review on Optimization of Fuzzy Controller Parameters in Robotic Applications. IETE J. Res..

[26] Peraza C., Ochoa P., Castillo O., Geem Z.W. (2022). Interval-Type 3 Fuzzy Differential Evolution for Designing an Interval-Type 3 Fuzzy Controller of a Unicycle Mobile Robot. Mathematics.

[27] Jiang Y., Yin S., Dong J., Kaynak O. (2020). A Review on Soft Sensors for Monitoring, Control and Optimization of Industrial Processes. IEEE Sens. J..

[28] Bradley E., Kantz H. (2015). Nonlinear time-series analysis revisited. Chaos Interdiscip. J. Nonlinear Sci..

[29] Benmouiza K., Cheknane A. (2013). Forecasting hourly global solar radiation using hybrid k-means and nonlinear autoregressive neural network models. Energy Convers. Manag..

[30] Long D., Zhang R., Mao Y. (2019). Recurrent Neural Networks With Finite Memory Length. IEEE Access..

[31] Ji W., Chan C. (2011). Prediction of hourly solar radiation using a novel hybrid model of ARMA and TDNN. Solar Energy.

[32] Taherdangkoo R., Tatomir A., Taherdangkoo M., Qiu P., Sauter M. (2020). Nonlinear Autoregressive Neural Networks to Predict Hydraulic Fracturing Fluid Leakage into Shallow Groundwater. Water.

[33] Kumar A., Irsoy O., Su J., Bradbury J., English R., Pierce B., Ondruska P., Gulrajani I., Socher R. Ask Me Anything: Dynamic Memory Networks for Natural Language Processing. Proceedings of the International conference on machine learning.

[34] Young T., Hazarika D., Poria S., Cambria E. (2018). Recent Trends in Deep Learning Based Natural Language Processing. IEEE Comput. Intell. Mag..

[35] Kalimuthu M., Mogadala A., Mosbach M., Klakow D. (2021). Fusion Models for Improved Image Captioning. ICPR International Workshops and Challenges, ICPR 2021, Lecture Notes in Computer Science.

[36] Yassin I.M., Zabidi A., Salleh M.K.M., Khalid N.E.A. Malaysian tourism interest forecasting using nonlinear auto regressive (NAR) model. Proceedings of the 3rd International Conference on System Engineering and Technology.

[37] Raturi R., Sargsyan H. (2018). A Nonlinear Autoregressive Scheme for Time Series Prediction via Artificial Neural Networks. J. Comput. Commun..

[38] Ahmed A., Khalid M. A Nonlinear Autoregressive Neural Network Model for Short-Term Wind Forecasting. Proceedings of the 2017 9th IEEE-GCC Conference and Exhibition (GCCCE).

[39] MATLAB 2022b (2022). Deep Learning Toolbox Reference.

[40] Padilla C., Hashemi R., Mahmood N., Latva-aho M. A Nonlinear Autoregressive Neural Network for Interference Prediction and Resource Allocation in URLLC Scenarios. Proceedings of the 2021 International Conference on Information and Communication Technology Convergence (ICTC).

[41] Adedeji P.A., Akinlabi S.A., Ajayi O.O., Madushele N. (2019). Non-Linear Autoregressive Neural Network (NARNET) with SSA filtering for a university Campus Energy Consumption Forecast. Procedia Manuf..

[42] Olney B., Mahmud S., Karam R. Efficient Nonlinear Autoregressive Neural Network Architecture for Real-Time Biomedical Applications. Proceedings of the 2022 IEEE 4th International Conference on Artificial Intelligence Circuits and Systems (AICAS).

[43] Li M., Ji S., Liu G. (2018). Forecasting of Chinese E-Commerce Sales: An Empirical Comparison of ARIMA, Nonlinear Autoregressive Neural Network, and a Combined ARIMA-NARNN Model. Math. Probl. Eng. Vol..

[44] Kummong R., Supratid S. (2019). Long-term forecasting system using wavelet – nonlinear autoregressive neural network conjunction model. J. Model. Manag..

[45] Davood N.K., Goudarzi G.R., Taghizadeh R., Asumadu-Sakyi A.B., Fehresti-Sani M. (2021). Long-term effects of outdoor air pollution on mortality and morbidity–prediction using nonlinear autoregressive and artificial neural networks models. Atmos. Pollut. Res..

[46] Domaschenko D., Nikulin E. (2017). Forecasting time series of the market indicators based on a nonlinear autoregressive neural network. Stat. Econ. Vol..

[47] Saba A.I., Elsheikh A.H. (2020). Forecasting the prevalence of COVID-19 outbreak in Egypt using nonlinear autoregressive artificial neural networks. Process. Saf. Environ. Prot..

[48] Newman E.I. (1988). Mycorrhizal links between plants: Their functioning and ecological significance. Adv. Ecol. Res..

[49] Bahram M., Põlme S., Kõljalg U., Tedersoo L. (2010). A single European aspen (Populus tremula) tree individual may potentially harbour dozens of Cenococcum geophilum ITS genotypes and hundreds of species of ectomycorrhizal fungi. FEMS Microbiol. Ecol..

[50] Schimel J.P., Bennett J. (2004). Nitrogen mineralization: Challenges of a changing paradigm. Ecology.

[51] Averill C., Turner B.L., Finzi A.C. (2014). Mycorrhiza-mediated competition between plants and decomposers drives soil carbon storage. Nature.

[52] Dickie I.A., Koele N., Blum J.D., Gleason J.D., McGlone M.S. (2014). Mycorrhizas in changing ecosystems^,^. Botany.

[53] Redecker D., Kodner R., Graham L.E. (2000). Glomalean Fungi from the Ordovician. Science.

[54] Humphreys C.P., Franks P.J., Rees M., Bidartondo M.I., Leake J.R., Beerling D.J. (2010). Mutualistic mycorrhiza-like symbiosis in the most ancient group of land plants. Nat. Commun..

[55] Lang C., Seven J., Polle A. (2010). Host preferences and differential contributions of deciduous tree species shape mycorrhizal species richness in a mixed Central European forest. Mycorrhiza.

[56] Simard S.W., Baluska F., Gagliano M., Witzany G. (2018). Mycorrhizal Networks Facilitate Tree Communication, Learning, and Memory. Memory and Learning in Plants.

[57] Castro-Delgado A.L., Elizondo-Mesén S., Valladares-Cruz Y., Rivera-Méndez W. (2020). Wood Wide Web: Communication through the mycorrhizal network. Tecnol. Marcha J..

[58] Beiler K.J., Simard S.W., Durall D.M. (2015). Topology of tree-mycorrhizal fungus interaction networks in xeric and mesic Douglas-fir forests. J. Ecol..

[59] Simard S.W., Asay A., Beiler K., Bingham M., Deslippe J., He X., Philip L., Song Y., Teste F., Horton T. (2015). Resource Transfer Between Plants Through Ectomycorrhizal Fungal Networks. Mycorrhizal Networks. Ecological Studies.

[60] Gorzelak M.A., Asay A.K., Pickles B.J., Simard S.W. (2015). Inter-plant communication through mycorrhizal networks mediates complex adaptive behaviour in plant communities. AoB Plants.

[61] Carreon H., Valdez F., Castillo O. (2022). A New Discrete Mycorrhiza Optimization Nature-Inspired Algorithm. Axioms.

[62] Liu P., Elaydi S.N. (2001). Discrete Competitive and Cooperative Models of Lotka–Volterra Type. J. Comput. Anal. Appl..

[63] Muhammadhaji A., Halik A., Li H. (2021). Dynamics in a ratio-dependent Lotka–Volterra competitive-competitive-cooperative system with feedback controls and delays. Adv. Differ. Equ..

[64] Din Q. (2013). Dynamics of a discrete Lotka-Volterra model. Adv. Differ. Equ..

[65] Liu X. (2010). A note on the existence of periodic solutions in discrete predator–prey models. Appl. Math. Model..

[66] Zhou Z., Zou X. (2003). Stable periodic solutions in a discrete periodic logistic equation. Appl. Math. Lett..

[67] Krabs W. (2003). A General Predator-Prey Model. Math. Comput. Model. Dyn. Syst..

[68] Allen L.J.S. (2007). An Introduction to Mathematical Biology.

[69] Brauer F., Castillo-Chavez C. (2012). Mathematical Models in Population Biology and Epidemiology.

[70] Müller J., Kuttler C. (2015). Methods and Models in Mathematical Biology, Deterministic and Stochastic Approaches. Lecture Notes on Mathematical Modelling in the Life Sciences.

[71] Voroshilova A., Wafubwa J. (2020). Discrete Competitive Lotka–Volterra Model with Controllable Phase Volume. Systems.

[72] Saha P., Bairagi N., Biswas M., Mondaini R. (2018). On the Dynamics of a Discrete Predator-Prey Model. Trends in Biomathematics: Modeling, 337 Optimization and Computational Problems.

[73] Zhao M., Xuan Z., Li C. (2016). Dynamics of a discrete-time predator-prey system. Advances in Difference Equations 2016.

[74] Chou C.S., Friedman A. (2016). Introduction to Mathematical Biology, Modeling, Analysis, and Simulations. Springer Undergraduate Texts in Mathematics and Technology.

[75] Raffoul Y.N. (2018). Qualitative Theory of Volterra Difference Equations.

[76] Bodine S., Lutz D.A. (2015). Asymptotic Integration of Differential and Difference Equations.

[77] Honda T., Iwata Y., Elaydi S., Hamaya Y., Matsunaga H., Pötzsche C. (2017). Operator Theoretic Phenomena of the Markov Operators which are Induced by Stochastic Difference Equations. Advances in Difference Equations and Discrete Dynamical Systems. ICDEA 2016.

[78] Mickens R.E. (2015). Difference Equations Theory, Applications and Advanced Topics.

[79] Kitzing K., Picard R., Siegmund S., Trostorff S., Waurick M., Elaydi S., Pötzsche C., Sasu A. (2019). A Hilbert Space Approach to Difference Equations. Difference Equations, Discrete Dynamical Systems and Applications, ICDEA 2017.

[80] Castro J.R., Castillo O., Melin P., Rodríguez-Díaz A. (2008). Building Fuzzy Inference Systems with a New Interval Type-2 Fuzzy Logic Toolbox. Transactions on Computational Science I.

[81] Chai T., Draxler R.R. (2014). Root mean square error (RMSE) or mean absolute error (MAE)?– Arguments against avoiding RMSE in the literature. Geoscientific Model Development..

[82] Saeed W., Ghazali R. (2016). Chaotic Time Series Forecasting Using Higher Order Neural Networks. Int. J. Adv. Sci. Eng. Inf. Technol..

[83] Martínez-García J.A., González-Zapata A.M., Rechy-Ramírez E.J., Tlelo-Cuautle E. (2022). On the prediction of chaotic time series using neural networks. Chaos Theory Appl..

[84] López-Caraballo C.H., Salfate I., A Lazzús J., Rojas P., Rivera M., Palma-Chilla L. (2016). Mackey-Glass noisy chaotic time series prediction by a swarm-optimized neural network. J. Physics: Conf. Ser..

